# A contribution to set a legal framework for biofertilisers

**DOI:** 10.1007/s00253-014-5828-y

**Published:** 2014-06-06

**Authors:** E. Malusá, N. Vassilev

**Affiliations:** 1 Research Institute of Horticulture, ul. Pomologiczna 18, 96-100 Skierniewice, Poland; 2 CRA-Centre for Plant–Soil Systems, Unit of Turin, Via Livorno 60, 10141 Turin, Italy; 3 University of Granada, c/Fuentenueva s/n, Granada, Spain

**Keywords:** PGPR, Mycorrhizal fungi, Rhizosphere, Regulation, Production standards

## Abstract

The extensive research, production and use of microorganisms to improve plant nutrition have resulted in an inconsistent definition of the term “biofertiliser” which, in some cases, is due to the different microbial mechanisms involved. The rationale for adopting the term biofertiliser is that it derives from “biological fertiliser”, that, in turn, implies the use of living microorganisms. Here, we propose a definition for this kind of products which is distinguishing them from biostimulants or other inorganic and organic fertilisers. Special emphasis is given to microorganism(s) with multifunctional properties and biofertilisers containing more than one microorganism. This definition could be included in legal provisions regulating registration and marketing requirements. A set of rules is also proposed which could guarantee the quality of biofertilisers present on the market and thus foster their use by farmers.

## Introduction

The development and use of microbial-based fertilisers has been increasing worldwide due to the recognition of the deleterious effects on the environment generated by the excessive and/or improper application of chemical fertilisers and of the improved knowledge about the relationships between the plant and all soil microorganisms occurring in the rhizosphere. Such potential has also prompted efforts in isolating and selecting microbial strains showing plant growth-promotion capabilities through direct and/or indirect improvement of plant nutrient uptake. Therefore, the positive agronomical effect of microbial-based products, besides products based on rhizobia species which have been successfully marketed since the 1950s, has opened a worldwide market of a new kind of fertilisers: the biofertilisers.

The different kinds of microorganisms utilised to improve plant nutrition (fungi or bacteria) and the different mechanisms utilised by them to obtain this final effect have created some inconsistencies in the definition of the biofertilisers, which has also been fuelled by the use of different wordings to name such products, frequently considered, rather inappropriately, as synonyms. This has created some confusion in the market of microbial-based products for plant nutrition, which in the EU has not been regulated yet. The present paper is aiming at providing some suggestions and issues to be considered by policy makers and industrial stakeholders in the process of development of a new legal provision, which should consider also the recently acquired scientific knowledge on microbial species beneficial for plant growth.

## Technical definition of biofertilizer

The term “biofertiliser” has been defined in different ways during the past 20 years, which derives from the improved understanding of the relationships occurring between the rhizosphere microorganisms and the plant. Okon and Labandera-Gonzalez (1994) were firstly arguing that rhizospheric organisms which improve soil nutrients utilisation but do not replace soil nutrients (such as mycorrhizal fungi or plant growth -promoting rhizobacteria (PGPR)) should not be called biofertilisers. Successively, focusing mainly on PGPR, Vessey (2003) proposed that the term biofertiliser should be associated to “a substance which contains living microorganisms which, when applied to seed, plant surfaces, or soil, colonizes the rhizosphere or the interior of the plant and promotes growth by increasing the supply or availability of primary nutrients to the host plant”. The term biofertiliser was derived from the contraction of the term biological fertiliser, with *biological*, implying the use of living organism.

Fuentes-Ramirez and Caballero-Mellado (2005) later defined a biofertilizer as “a product that contains living microorganisms, which exert direct or indirect beneficial effects on plant growth and crop yield through different mechanisms”. They widened the definition, extending it to also include bacteria used to control plant pathogens. Nevertheless, microorganisms which promote plant growth by control of harmful organisms, such as biofungicides, bionematocides, bioinsecticides, or any other products with similar activity favoring plant health, are generally defined as biopesticides, not as biofertilisers (Siddiqui and Mahmood 1999; Vessey 2003).

The principal mechanism of action of a microorganism in enhancing plant growth has prompted the proposition of calling them with terms different from biofertilisers. Those that can enhance plant growth by producing phytohormones are regarded as bioenhancers or phytostimulators, while those degrading organic pollutants which can impair plant growth are named rhizoremediators (Somers et al. 2004). However, even though the principal mechanism expressed by a species could characterise its classification, in most studied cases, and particularly under field conditions, a single microorganism will often reveal multiple mechanisms of action (Kloepper 1993; Vessey 2003), thus making further sub-classification a merely theoretical exercise.

Frequently, scientific literature refers to the term biofertiliser as a simple microorganism showing plant growth promotion effects (see several references in Bardi and Malusá 2012). However, for it to be used within agronomical practices, any beneficial microorganism requires to be included in formulations that allow its storage in the timeframe from production, till its field application, and effective delivery to the soil or plant. It derives that the term ‘biofertiliser’, in analogy to the chemical or organic fertilisers, should refer to a product that is ready to be commercialised, thus composed of beneficial strain/s included in a carrier, also with the possible inclusion of additives that could increase the efficacy of the microorganism’s activity. Therefore, whether the microorganism increases the growth of plants by replacing soil nutrients (i.e. by biological N_2_ fixation), by making nutrients more available to plants (i.e. by solubilisation of nutrients) or by increasing plant access to nutrients (i.e. by increasing the volume of soil accessed by the root system), as long as the nutrient status of the plant has been enhanced by the microorganism (Vessey 2003), it is the *formulated* product containing the microorganisms that is applied to the plant or soil that shall be named biofertiliser.

It derives that a biofertiliser shall not be used for an organic and/or mineral fertiliser and that the term ‘biofertiliser’ shall not be used interchangeably or as a synonym with the wordings defining different kinds of organic fertilizers (e.g. compost, plant extracts) or biostimulants derived from microorganisms (e.g. products containing dead microbial cells, microbial culture extracts, microbial cell extracts, etc.).

The need of a legal definition for biofertilisers derives also by the presence of descriptions that could lead to erroneous classifications: For example, mycorrhizal fungi were indicated as “a natural part of plants” (Gianinazzi and Vosatka 2004) and not as microorganisms, which would result, if accepted, in avoidance of complicated (and expensive) registration procedures but could also open the market to fraudulent products.

## Examples of legal definitions of biofertilisers

The legal definition of a marketable product such as the biofertiliser is key for producers willing to commercialise them. In the European Union (EU) and in the USA, there are currently no legal definitions for the term ‘biofertiliser’, or specific legal provisions defining their characteristics. In the EU, microorganisms (bacteria, viruses and fungi) are included as possible inputs in the EU Commission Regulation n. 889/2008 on organic production, but only for the biological control of pests and diseases. As such, they are thus listed within the legal framework dealing with plant protection products, as biocontrol agents. Similarly, the US National Organic Program foresees only the possibility of using biological organisms for plant protection.

The drawbacks deriving from the lack of a specific legal definition for the biofertilisers can be highlighted considering the case of products containing arbuscular mycorrhizal fungi (AMF). In 1999, a product containing AMF and claiming only biostimulating and growth-promotion properties was not classified as a plant protection product by the EU General Directorate for Health and Consumers (DG SANCO). However, in 2003 and 2008, two other products based on AMF, but claiming action against fungi or protection of the root system, were assessed as biological control agents, thus requiring registration according to the rules established for biological control substances (i.e. EU Regulation (EC) 1107/2009).

India is probably the country with the most complete legal framework related to biofertilisers. The Indian Ministry of Agriculture issued an order in 2006, later amended in 2009, which included biofertilisers under the Essential Commodities Act of 1955, and within the order for the control of fertilizers of 1985. In this act, the term biofertiliser means “the product containing carrier based (solid or liquid) living microorganisms which are agriculturally useful in terms of nitrogen fixation, phosphorus solubilization or nutrient mobilization, to increase the productivity of the soil and/or crop”. The term is also covered under the broad definition of fertilisers, which “means any substance used or intended to be used as a fertilizer of the soil and/ or crop”.

Considering the trend of the policies in developed countries for a more sustainable and environmentally friendly agricultural activity (e.g. EU COM (2011) 21; EU COM (2011) 627; EU COM (2010) 2020 final) and the globalisation of the markets for microbial-based fertilisers, there is a need of developing adequate standards and legal provisions to support the production and use of biofertilisers (i.e. formulated products containing microorganisms). This could be initially developed at international level through the ISO Standards, updating the currently available standards for fertilisers and soil conditioners (e.g. ISO Standard 7851:1983 on Fertilizers and soil conditioners—Classification and ISO Standard 8157:1984 on Fertilizers and soil conditioners—Vocabulary) or by developing a new standard under the ISO Technical Committee 276 on Biotechnology.

## Legal quality of biofertilisers

The quality of a biofertiliser shall be assured in order to guarantee the success of the inoculation and to promote acceptance by the farmers. Normally, the term ‘quality’ refers only to the density and viability of the available microorganisms and their preservation (Herridge and Peoples 1990; Somasegaran and Hoben 1994).

However, to assure a proper quality of the product for the final users, the legislation should introduce other parameters to be controlled at production level, which can later be reflected in the labelling requirements. The production standard and the label shall include the definition of parameters such as the microbial density at the time of manufacture and at the expiry date, the expiry period, the permissible contamination, the pH, the moisture, the identification of the microbial strain, and the specification of the kind of carrier utilised.

### The current situation in some EU member states

The lack of specific regulations in the European Union setting quality parameters for biofertilizers is leaving space to national or regional rules, which are not homogeneous. For example, the Polish Law on Fertilizers and Fertilization of July 10th 2007 includes “growth stimulators” in the category of plant conditioners. These are products which have “a positive impact on plant growth or other metabolic processes of plants in other ways than plant nutrients” and shall “pose no threat to [the] health of humans or animals or to the environment after their use according to use and storage instruction”. This definition can be applied to biofertilisers, but no specific requirements are foreseen for such category of products.

Spain, which is the second largest producer of conventional fruit and vegetables after Italy and among the leading countries in organic crops in Europe, does not include the term ‘biofertiliser’ in its legislation. The newest legal provision dealing with fertilizers (Real Decreto 506/2013) defines the number of microorganisms in organic amendments and compost but does not mention plant beneficial microorganisms. Fertilisers are defined as “Products used in agriculture or gardening, which for their nutrient content facilitate plant growth, increase performance and improve crop quality or which by their specific action, amending, as appropriate, modify soil fertility or its physical, chemical or biological properties and that meet the requirements of Article 4.2 of this Royal Decree characteristics.” Fertilisers, specialty products and amendments are also included in this definition. The Spanish administrative system allows local administrations to additionally regulate the matter: For example, the Local Government of Andalucía, the Spanish region with the highest organic crop production, has foreseen a category of products allowed to be used in organic farming called biofertilisers, which is formed by “a group of organisms that are applied to soil or seeds to improve plant nutrition (rhizobium, mycorrhiza, *Azotobacter*, etc.)”. However, this definition also includes “preparations derived from biological fermentation containing groups of nutrients that are used basically as foliar fertilizers” (Junta de Andalucía, http://www.juntadeandalucia.es).

In Italy, only the mycorrhizal fungi inocula are included within the group of “Products with action on the soil” and in the miscellaneous category of “Products with specific action” foreseen in the Decreto Legislativo 29 april 2010, n. 75. The quality requirements established by the legal provision foresee that the inoculum is reproduced under sterile conditions on roots of sorghum in a substrate formed by an organic soil conditioner and rhizosphere bacteria. These conditions, particularly the “sterile conditions” requirement, are practically very difficult to achieve, considering the need of organic substrate. Besides, the presence of rhizosphere bacteria is, from the point of view of the mycorrhizal fungus, requiring an unsterile condition of the substrate. The label of such products shall indicate which organic matrix is used (presumably as a carrier), the name of the mycorrhizal fungus species included, and the name of rhizosphere bacteria and trichoderma species, even though the last two kinds of microorganisms are not AMF. No genetically modified organisms are allowed to be utilised for making this product; pathogens such as *Salmonella* spp., *Escherichia coli*, and other aerobic mesophilic microorganisms and nematode eggs shall not be present.

### The situation in other countries

Some countries where the biofertilisers industry has been strongly developed in the last years have already enacted some regulations. China has set their legal quality based on eight parameters: amount of living cells, carbon and water content, pH, size of granules (for solid products), appearance, contamination and validity (Suh et al. 2006). The amount of living cells is considered by the Chinese standard as the most important parameter for assessing the quality of the different kinds of biofertilisers. Indeed, it has been defined for seven categories of microorganisms (rhizobia, distinguished between fast or slow growing species; N-fixing bacteria; phosphorous solubilising bacteria (PSB), separated for the capacity of acting on organic or inorganic P; Si-solubilising bacteria and multi-strain consortia). Depending on the kind of bacteria utilised for the production of the biofertiliser, the amount of living cells ranges between >0.5 × 10^9^ CFU mL^−1^ or >0.1 × 10^9^ CFU g^−1^ and >1.5 × 10^9^ CFU mL^−1^ or >0.2 × 10^9^ CFU g^−1^, for liquid and solid products, respectively. It is also required that the organic matter (C) content of the biofertiliser shall be at least 20 %, irrespective of the physical form and that the product has at least 6 months of validity (Suh et al. 2006).

In India, the decree on the control of fertilisers of 1985 enacted by the Ministry of Agriculture, as modified (Ministry of Agriculture 2009), prescribes production and marketing standards with regard to the different kinds of microorganisms forming the biofertiliser. The standard sets out seven quality parameters: the physical form, the minimum count of viable cells, the contamination level, pH, the particle size in case of carrier based materials, the maximum moisture percent by weight of carrier based products, and the efficiency character. Four groups of microorganisms are considered: *Rhizobium*, *Azotobacter*, *Azospirillum*, phosphate-solubilising bacteria and mycorrhizal fungi. In case of bacteria, the minimum count of viable cells is 5 × 10^7^ cells per gram of solid carrier, or 1 × 10^8^ cells per ml of liquid carrier. For products containing mycorrhizal fungi, at least 100 viable propagules must be present per gram of finished product. The efficiency in fixing nitrogen must be shown with different tests: Rhizobia shall show effective nodulation; *Azotobacter* strain shall be capable of fixing at least 10 mg N per g of sucrose consumed, while *Azospirillum* strains must be able to form a white pellicle in semisolid N-free bromothymol blue media. The activity of PSB can be assessed spectrophotometrically (30 % P solubilisation) or by the formation of a solubilisation zone of at least 5 mm in a media having at least 3 mm thickness. Products with mycorrhizal fungi shall be able to provide 80 infection points in roots per gram of inoculum used. For each group of microorganisms, a detailed procedure for the quality control is also specified.

## Proposals for an EU legislation on biofertilisers

The overall EU policy for the development of the agricultural sector in the next programming period (EU COM (2011)) underlines the need of reducing the impact on the environment of agricultural practices and the possibility of an increased use of alternatives to chemical inputs. The achievement of the objectives of rural development, which contribute to the Europe 2020 strategy for smart, sustainable and inclusive growth (EU COM (2010)), shall be pursued, among others, through the improvement of soil management, the preservation of biodiversity, the fostering of knowledge transfer and innovation and the promotion of resource efficiency. Furthermore, there is a strong emphasis on a wider application of agricultural practices based on low input (e.g. EU Directive 2009/128 on the sustainable use of pesticides) and on organic farming practices. Based on these policies, the support to research dedicated to biotechnological processes and products has a strong focus through the Horizon 2020 Programme (EU COM (2011) 808). In such a context, it is thus feasible to expect an increased interest among producers to develop products based on biological compounds and microorganisms.

On the other hand, an economically interesting share of the fertilisers’ market is already allocated to nitrogen fixing biofertilisers, phosphate-solubilising biofertilisers, potash-mobilising biofertilisers and other biofertilisers like zinc and sulphur-solubilising biofertilisers. Nevertheless, major restraints of the industry derive from the lack of awareness about the concept of biofertilisers, low rate of adoption by the farmers and presence in the market of low-quality products that are disturbing its development. It would thus be important to define a legal framework on biofertilisers which should protect both the reliable manufacturers of biofertilisers and the farmers utilising an effective product from a market which allows low-quality products. In this respect, it is important to consider a legal definition for biofertilisers that would include all microorganisms currently used, but would also allow the widening of the scope to new categories (e.g. protozoa).

A biofertiliser could thus be defined as the formulated product containing one or more microorganisms that enhance the nutrient status (and the growth and yield) of the plants by either replacing soil nutrients and/or by making nutrients more available to plants and/or by increasing plant access to nutrients.

### Quality standards for production and marketing of biofertilizers

The quality standard of biofertilisers shall include parameters which can be listed in the label or that will be required to be provided in the dossier for the authorisation for marketing. We consider essential to foresee: the minimum count of viable cells/propagules, the efficiency in nutrient solubilisation and or fixation (in case of bacteria), the efficiency in plant inoculation (with respect to mycorrhizal fungi), the validity (shelf-life and/or expiry date), the contamination level, the pH, the physical form, carbon and water content (Table 1). For the minimum count of viable cells/propagules and for the efficiency data, a range of values could be established, considering the technical possibilities from the manufacturers’ side and the data from researches. The correct identification of the PGPM strain included in the commercial preparations is also necessary. This shall be carried out according to molecular biology methods and the distinguishing characteristics of the strain provided in the registration dossier.

**Table 1:** Summary of proposed features to be included in a legal provision regulating the production and marketing of biofertilisers in EU

Requirement	Item	Proposal
Definition of biofertiliser		The formulated product containing one or more microorganisms that enhance the nutrient status (the growth and yield) of the plants by either replacing soil nutrients and/or by making nutrients more available to plants and/or by increasing plant access to nutrients
Quality parameters for registration purposes	a. Composition	List of strains included in the product and minimum amount in percent (weight or volume) for each category of PGPM (bacteria, fungi, etc.). The information should include whether or not the strain is registered and in a positive case in which collection/country.
	b. Strain characteristics	Molecular characterisation of the strains used
	c. Additives or other substances	List of ingredients and their amount
	d. Minimum count of viable cells/propagules	A range of values (colony-forming units per gram) valid for the different kinds of PGPM present in the product
	e. Physical form	Solid, liquid, gel, emulsion, etc.
	f. pH	–
	g. C content	Total C
	h. Water content	%
	i. Efficiency in nutrient solubilisation	In case of either bacteria or fungi determined under laboratory conditions
	j. Efficiency in N fixation	For N fixing microorganisms determined under laboratory conditions
	k. Efficiency in plant inoculation	For mycorrhizal fungi (e.g. minimum percentage of root host inoculation points under defined conditions) and endophytic microorganisms
	l. Contamination level	Amount of possible contaminants (e.g. pathogen microorganisms)
	m. Validity	Shelf-life and/or expiry date
	n. Storage conditions	Method of storage
Evaluation dossier	Efficacy studies	Set of studies (reports, scientific papers) demonstrating the efficacy of the product for the crop/s under field and/or greenhouse conditions, with the doses and application methods specified in the label. Possibly to be carried out according to standards similar to Good Experimental Practices (GEP) adopted by the EU for analogous purposes
	Eco-toxicology and toxicology studies	Not compulsory; required only in case of possible risk due to specific additives or strains potentially pathogens for human
Data to be included in the label of the product	Data mentioned in points *a*, *d*–*k*, *m*–*n* of quality parameters for registration purposes	Same information as above
	Target crop/s	Name of crop/s
	Application method/s	Description of the method (e.g. broadcasting over the soil surface, in-furrow application, dry dusting or slurring of seeds, spraying, etc.)
	Doses and the timing	Period or the number of applications during the season
	Additional information or precautions	Any additional information that would be useful for efficient use of the product (e.g. soil management practices, chemical fertilisation, spraying nozzles size, sprayer pressure, etc.)

It is considered important to require the manufacturers to define and include in the label the target crop/s and application method/s (Table 1). Indeed, seeing that the application of biofertilisers is a new practice for the majority of farmers, it is crucial they are supported with such kind of instruction in order to increase their awareness on the issues favouring the efficiency of the application in the field (size of the inoculated microbial population and closeness to the target organ of the crop plant), which can reduce the likelihood of misuse or of an unsatisfactory effect of the inoculation and of inconsistent results. Therefore, indication about the method of application (e.g. broadcasting the biofertiliser over the soil surface or by in-furrow application, as well as dry dusting or slurring of seeds, and distributing through the fertigation system or spraying), the doses and the timing (either the period or the number of applications during the season) should be provided in the label. The latter information is particularly needed in case of vegetable and fruit crops. Recovery of the inoculated strains in the soil or on root rhizosphere for plant growth-promoting bacteria was limited to 30–40 days after inoculation (Bashan et al. 1995). Therefore, repeated applications (three to four times) during the growing season would increase the effectiveness of application. Additional information for liquid products, regarding the sprayer parameters, could also be considered. Indeed, bacteria viability was found to be affected by the length of spraying time (Świechowski et al. 2012). Orchard sprayers, both with pneumatic and hydraulic atomisation systems, operated at standard application parameters, were feasible to maintain a good viability of *Pseudomonas fluorescens* and *Enterobacter nimipressuralis* strains. However, in a 2-h test simulating the worst case application parameters, the viability of sprayed bacteria was reduced to about 90 % for *P. fluorescens* and 82 % for *E. nimipressuralis* (Świechowski et al. 2012).

The instructions for the farmer concerning the application of the biofertiliser should also contain a reference on the soil management practices, particularly on the reduction of the quantity of chemical fertilisers applied. Indeed, higher efficacy of colonisation and activity of PGPM is expressed under low nutritional conditions (e.g. reduction by 20–50 % of chemical fertilisers has been proved feasible with several crops) (Adesemoye et al. 2008, 2009; Jeffries et al. 2003) and an efficient mycorrhizal symbiosis can substitute up to 222 kg P_2_O_5_ ha^−1^ (Kelly et al. 2001). However, a reference to weed control and irrigation practices could also be concerned, since both of them can affect the survival of inoculated microorganisms (Watt et al. 2006).

The implementation of ISO standards during the production process (e.g. ISO 9000:^2005^) could be an additional, voluntary, requirement providing assurance about the product and its quality and giving an added marketing value to the product.

### Additives and carriers to biofertilisers

The provision should also allow and define the role of stimulatory compounds or additives included in the formulation aiming at increasing the efficiency of inoculation (Table 1). Indeed, it has been proved that root inoculation by AMF was increased by humic acids or other organic fractions (Gryndler et al. 2005), and supplementing with chitin or chitin-containing materials enhanced bacteria-induced plant growth (Manjula and Podile 2001). Therefore, the addition of substances having plant-beneficial effect should be proved at the conditions of the biofertiliser application into the target soil–plant system. In this respect, we believe that only microbial-based fertilisers, where the principal function is due to the microbial species forming the inoculum, should be included in the category of biofertilisers; when the microorganism/s are only an ingredient of a product based mainly on other substances (either inorganic or organic), it should not be included into such category. The rationale to this is similar to that applied for plant protection products, where it is the active substance that defines the kind of commercial product, and not the additives used in the formulation to allow its distribution or even increase the efficacy of the active substance itself.

The selection of the carrier for the inoculant is crucial to support both the marketing of the inoculant and the delivery of a suitable amount of PGPM in good physiological condition (Smith 1992; Malusá et al. 2012). For this reason, besides the common inorganic, organic and polymeric compounds used, a new biological approach in carrier production is currently under development with the use of bacterial biofilms or nanocarriers (Jayasinghearachchi and Seneviratne 2004; Qureshi et al. 2005; Seneviratne et al. 2007). The use of nanomaterials in food-related productions has been reviewed by Magnuson et al. (2011), which mentioned nanomaterials used in agricultural production as sources of unintentional nanomaterials in foods. Considering the current policy and guidelines for the assessment of the health risks derived from their utilisation (Scientific Committee EFSA 2011), it would be advisable to foresee already in the legislation some quality standards. Indeed, in 2012 the International Center for Technology Assessment petitioned the Environmental Protection Agency (EPA) in the USA to examine three registration applications for a pesticide containing micronised copper carbonate, which would be considered a nanoscale material. EPA has taken a position that all nanoscale materials in pesticides are considered to be ‘new’ and must undergo a full review even if they have been previously approved. Furthermore, in 2012, the Council of Europe Parliamentary Assembly began the first steps towards nanotechnology regulation with a view to respecting the scientific precautionary principles.

### Evaluation of biofertilisers

The data to be provided by manufacturers during the registration process shall include information about the efficacy of the product in terms of either plant nutrition or growth or yield (Table 1). This kind of data is normally required by current national legal provisions for any kind of fertiliser and thus should not be a major concern for producers. However, the report or scientific article describing the effect of the biofertiliser shall define the conditions and crop/s object of the trial to allow a proper evaluation of the product’s efficacy and of the instructions for its application.

The evaluation of the efficacy of biofertilisers based on microorganism consortia could be more difficult to perform. A consortium containing a mixture of PGPM, stimulating plant growth at different growth stages, could show more mechanisms of action, sometimes overlapping also plant protection mechanisms. Indeed, it has been proved that consortia of species normally improving nutrient efficiency (e.g. double inoculants with PSM + AMF, or *Rhizobium* + AMF in one gel-formulation) can also show plant protection properties (e.g. Vassilev et al. 2001, 2006a, b). Furthermore, farmers like to utilise “multifunctional” products and manufacturers prefer to market products with several activities because they are more likely to have effects and attract users. For this reason, to avoid the registrations of biofertilizers that would later be marketed for their additional (biocontrol) effects, a clear discrimination shall be posed in the legal provision among the possible claims advertised by the product on the label. The principal function of the product would thus lead to its classification, even if others can be performed.

Nevertheless, better nutrient efficacy has been reported in case of several kinds of consortia: mixtures of *Rhizobium* sp. and PGPR (Sivaramaiah et al. 2007), associations of nodule-inducing rhizobia, free-living N-fixing bacteria and AM fungi (Adesemoye et al. 2008; Barea et al. 2002; Lisette et al. 2003; Toro et al. 1998; Wang et al. 2011; Wu et al. 2005), formulations of different categories of bacteria such as *Rhizobium* and PSB (Alagawadi and Gaur 1988), PGPR, PSB and *Rhizobium* (Prasad and Chandra 2003), PSB and KSB (Han and Lee 2005; Vassilev et al. 2006), as well as different combinations of PGPR and AMF (Malusá et al. 2007; Singh and Adholeya 2003). Therefore, there should not be difficult to exploit these kinds of consortia for fertilisation purposes.

Normally, the strains employed in the production of biofertilisers are derived from natural conditions (i.e. isolated from the soil or the rhizosphere), therefore there should not be a specific environmental risk deriving from their application. However, the registration should provide sufficient data to evaluate possible risks about eco-toxicology and fate in the environment particularly when additives are included in the formulation.

The provision of toxicological data should not be considered a standard requirement necessary for registration purposes. However, in case strains of PGPM species utilised in the formulation are known to be potentially or opportunistic pathogens for mammals (e.g. *Enterobacter*, *Pantoea*, *Klebsiella* and *Burkholderia*), such kind of data shall be required to be provided and assessed before granting the authorisation for marketing (Frangolias et al. 1999; Guo et al. 2002).

## Conclusions

The global market for biofertilisers in terms of revenue was estimated to be worth about 5 billion USD in 2011 and, according to a detailed analysis of the current market and of the scenarios for its development in the different continents, is forecasted to double by 2017 (Marketsandmarkets 2013). Latin America is among the currently top consumers of biofertilisers: In Mexico, a program to support the introduction of N-fixing biofertilizers based on *Azospirillum* was carried on 1.5 million hectares (Fuentes-Ramirez and Caballero-Mellado 2005). According to estimates of the Indian National Bio-fertilizer Development Center (NBDC) and the Bio-Tech Consortium of India Ltd (BCIL), about 350.000–500.000 tonnes of biofertilisers are potentially required for Indian agriculture (Dewasthale and Bondre 2008).

Nevertheless, additional efforts are required to be addressed when dealing with manufacturing biofertilisers. The formulation of inocula and the possibility of developing a ‘universal’ PGPM inoculant for each important field crop could further foster the use of biofertilisers. Use of strains cooperating with autochthonous microorganisms, such as endophytic bacteria (Reinhold-Hurek and Hurek 2011; Ryan et al. 2008), or other categories of microorganisms such as yeasts (El-Tarabily and Sivasithamparam 2006), unculturable bacteria species, as well as the inclusion of protozoa in the formulation of biofertilisers (Bonkowski 2004; Knox et al. 2003; Ronn et al. 2002) could also be key for the development of new kinds of biofertilisers. Finally, the addition of biological substances to the formulation improving the colonisation rate or the survival of the inocula, such as the strigolactones synthetic analogs in the AM fungi–plant symbiosis (Xie et al. 2010), can also be soon transferred into biofertiliser manufacturing technologies. A new legal provision shall anticipate such aspects, providing at least guidelines or principia to be followed by the manufacturers.

A final consideration could include the approach in evaluating the efficacy of biofertilisers. It was pointed out that the “variability and inconsistency” of field results after inoculation with PGPM is being based on the comparison of experiments carried out under different environmental conditions, with different cultivars, in soils with different characteristics, and not taking into account others factors related to the inocula (Fuentez-Ramirez and Caballero-Mellado 2005). However, a similar degree of variability in the results can be observed with the application of mineral or organic fertilisers, when considering the effect of the same range of external factors. The application of biofertilisers shall thus be considered in the framework of the farming system in which they are applied, favouring synergistic interactions with different agronomical practices affecting soil’s physical and chemical conditions (such as pH, water availability, salinity, organic matter content) such as minimum tillage or precision agriculture, irrigation and pests control. The designing of biofertilisation programs by providing different consortia, each with specific nutritional features best suited for the different phenological phases of the crop, similarly to what is currently done with chemical fertilizers, would also improve their use.

## References

[CR1] Adesemoye AO, Torbert HA, Kloepper JW (2008). Enhanced plant nutrient use efficiency with PGPR and AMF in an integrated nutrient management system. Can J Microbiol.

[CR2] Adesemoye AO, Torbert HA, Kloepper JW (2009). Plant growth-promoting rhizobacteria allow reduced application rates of chemical fertilizers. Microb Ecol.

[CR3] Alagawadi AR, Gaur AC (1988). Associative effect of *Rhizobium* and phosphate solubilizing bacteria on the yield and nutrient uptake of chickpea. Plant Soil.

[CR4] Bardi L, Malusá E, Haryana N, Punj S (2012). Drought and nutritional stresses in plant: alleviating role of rhizospheric microorganisms. Abiotic stress: new research.

[CR5] Barea JM, Azcon R, Azcon-Aguilar C (2002). Mycorrhizosphere interactions to improve plant fitness and soil quality. Antonie Van Leeuwenhoek.

[CR6] Bashan Y, Puente EM, Rodriguez-Mendoza MN, Toledo G, Holguin G, Ferrera-Cerrato R, Pedrin S (1995) Survival of *Azospirillum brasilense* in the bulk soil and rhizosphere of 23 soil types. Appl Environ Microbiol 61:1938–1945 10.1128/aem.61.5.1938-1945.1995 PMC1388448 16535030

[CR7] Bonkowski M (2004). Protozoa and plant growth: the microbial loop in soil revisited. New Phytol.

[CR8] Decreto Legislativo 29 aprile 2010, n. 75 “Riordino e revisione della disciplina in materia di fertilizzanti, a norma dell'articolo 13 della legge 7 luglio 2009, n. 88” Gazzetta Ufficiale n.121 of 26-5-2010-Suppl. Ordinario n. 106

[CR9] Dewasthale G, Bondre (2008). Marketing of biofertilizers. Conference on Rural Marketing.

[CR10] EU Directive 2009/128/EC of The European Parliament and of the Council of 21 October 2009 establishing a framework for community action to achieve the sustainable use of pesticides. EU, Brussels

[CR11] El-Tarabily KA, Sivasithamparam K (2006). Potential of yeasts as biocontrol agents of soil-borne fungal plant pathogens and as plant growth promoters. Mycoscience.

[CR12] EU COM(2010) 2020 final (2010) Communication from the Commission Europe 2020: A strategy for smart, sustainable and inclusive growth. EU, Brussels

[CR13] EU COM(2011) 627 (2011) Support for rural development by the European Agricultural Fund for Rural Development (EAFRD). EU, Brussels

[CR14] EU COM(2011) 808: Horizon 2020 (2011) The Framework Programme for Research and Innovation. EU, Brussels

[CR15] EU Commission Regulation (EC) No 889/2008 of 5 September 2008 laying down detailed rules for the implementation of Council Regulation (EC) No 834/2007 on organic production and labelling of organic products with regard to organic production, labelling and control. EU, Brussels

[CR16] Frangolias DD, Mahenthiralingam E, Rae S, Raboud JM, Davidson AGF, Wittmann R, Wilcox PG (1999). *Burkholderia cepacia* in cystic fibrosis—variable disease course. Am J Respir.

[CR17] Fuentes-Ramirez LE, Caballero-Mellado J, Siddiqui ZA (2005). Bacterial biofertilizers. PGPR: biocontrol and biofertilization.

[CR18] Gianinazzi S, Vosatka M (2004). Inoculum of arbuscular mycorrhizal fungi for production systems: science meets business. Can J Bot.

[CR19] Gryndler M, Hršelová H, Sudová R, Gryndlerová H, Řezáčová V, Merhautová V (2005). Hyphal growth and mycorrhiza formation by the arbuscular mycorrhizal fungus *Glomus claroideum* BEG 23 is stimulated by humic substances. Mycorrhiza.

[CR20] Guo X, van Iersel MW, Chen J, Brackett RE, Beuchat LR (2002). Evidence of association of salmonellae with tomato plants grown hydroponically in inoculated nutrient solution. Appl Environ Microbiol.

[CR21] Han HS, Lee KD (2005). Phosphate and potassium solubilizing bacteria effect on mineral uptake, soil availability and growth of eggplant. Res J Agric Biol Sci.

[CR22] Herridge DF, Peoples MB (1990). Ureide assay for measuring nitrogen fixation by nodulated soybean calibrated by ^15^ N methods. Plant Physiol.

[CR23] International Organization for Standardization (ISO) Standard 9000:2005. Quality management systems - Fundamentals and vocabulary. ISO, Switzerland, pp 1–30

[CR24] Jayasinghearachchi HS, Seneviratne G (2004). A bradyrhizobial-*Penicillium* spp. biofilm with nitrogenase activity improves N_2_ fixing symbiosis of soybean. Biol Fertil Soils.

[CR25] Jeffries P, Gianinazzi S, Perotto S, Turnau K, Barea JM (2003). The contribution of arbuscular mycorrhizal fungi in sustainable maintenance of plant health and soil fertility. Biol Fertil Soils.

[CR26] Kelly RM, Edwards DG, Thompson JP, Magarey RC (2001). Responses of sugarcane, maize, and soybean to phosphorus and vesicular–arbuscular mycorrhizal fungi. Aust J Agric Res.

[CR27] Kloepper JW, Metting EB (1993). Plant-growth-promoting rhizobacteria as biological control agents. Soil microbial ecology: applications in agricultural and environmental management.

[CR28] Knox OGG, Killham K, Mullins CE, Wilson MJ (2003). Nematode-enhanced colonization of the wheat rhizosphere. FEMS Microbiol Lett.

[CR29] Lisette J, Xavier C, Germida JJ (2003). Selective interactions between arbuscular mycorrhizal fungi and *Rhizobium leguminosarum* bv. *viceae* enhance pea yield and nutrition. Biol Fertil Soils.

[CR30] Magnuson BA, Jonaitis TS, Card JW (2011). A brief review of the occurrence, use, and safety of food-related nanomaterials. J Food Sci.

[CR31] Malusá E, Sas-Paszt L, Zurawicz E, Popinska W (2007). The effect of a mycorrhiza-bacteria substrate and foliar fertilization on growth response and rhizosphere pH of three strawberry cultivars. Int J Fruit Sci.

[CR32] Malusá E, Sas-Paszt L, Ciesielska J (2012) Technologies for beneficial microorganisms inocula used as biofertilizers. The Scientific World Journal. doi:10.1100/2012/491206 10.1100/2012/491206 PMC3324119 22547984

[CR33] Manjula K, Podile AR (2001). Chitin-supplemented formulations improve biocontrol and plant growth promoting efficiency of *Bacillus subtilis* AF 1. Can J Microbiol.

[CR34] Marketsandmarkets (2013) Global biofertilizers market by types, applications and geography—trends and forecasts to 2017. Marketsandmarkets, Dallas TX, USA

[CR35] Ministry of Agriculture, Government of India. Biofertilizers and organic fertilizers covered in fertilizer (Control) Order, 1985 (as amended, March 2006 and November 2009). Official Gazette 3 November, 2009

[CR36] EU Regulation (EC) No 1107/2009 of the European Parliament and of the Council of 21 October 2009 concerning the placing of plant protection products on the market and repealing Council Directives 79/117/EEC and 91/414/EEC. EU, Brussels

[CR37] Okon Y, Labandera-Gonzalez CA (1994). Agronomic applications of *Azospirillum*: an evaluation of 20 years worldwide field inoculation. Soil Biol Biochem.

[CR38] Prasad H, Chandra R (2003). Growth pattern of urdbean *Rhizobium* sp. with PSB and PGPR in consortia. J Indian Soc Soil Sci.

[CR39] Qureshi N, Annous BA, Ezeji TC, Karcher P, Maddox IS (2005). Biofilm reactors for industrial bioconversion processes: employing potential of enhanced reaction rates. Microb Cell Factories.

[CR40] Reinhold-Hurek B, Hurek T (2011). Living inside plants: bacterial endophytes. Curr Opin Plant Biol.

[CR41] Ronn R, McGraig AE, Griffiths BS, Prosser JI (2002). Impact of protozoan grazing on the bacterial community structure in soil microcosms. Appl Environ Microbiol.

[CR42] Ryan RP, Germaine K, Franks A, Ryan DJ, Dowling DN (2008). Bacterial endophytes: recent developments and applications. FEMS Microbiol Lett.

[CR43] Scientific Committee EFSA (2011). Scientific opinion on guidance on the risk assessment of the application of nanoscience and nanotechnologies in the food and feed chain. EFSA J.

[CR44] Seneviratne G, Zavahir JS, Bandara WM, Weerasekara MLMAW (2007). Fungal–bacterial biofilms: their development for novel biotechnological applications. World J Microbiol Biotechnol.

[CR45] Siddiqui ZA, Mahmood I (1999). Role of bacteria in the management of plant parasitic nematodes. A review. Bioresour Technol.

[CR46] Singh R, Adholeya A (2003). Interactions between arbuscular mycorrhizal fungi and plant-growth promoting rhizobacteria. Mycorrhiza News.

[CR47] Sivaramaiah N, Malik DK, Sindhu SS (2007). Improvement in symbiotic efficiency of chickpea (*Cicer arietinum*) by coinoculation of *Bacillus strains* with *Mesorhizobium* sp. *Cicer*. Indian J Microbiol.

[CR48] Smith RS (1992). Legume inoculant formulation and application. Can J Microbiol.

[CR49] Somasegaran P, Hoben HJ (1994). Handbook for Rhizobia. Methods in legume-Rhizobium technology.

[CR50] Somers E, Vanderleyden J, Srinivasan M (2004). Rhizosphere bacterial signaling: a love parade beneath our feet. Crit Rev Microbiol.

[CR51] Real Decreto 506/2013, de 28 de junio, sobre productos fertilizantes. Spain Boletín Oficial del Estado N. 164. Sec. I.: 51119-51207

[CR52] Suh JS, Jiarong P, and Toan PV (2006) Quality control of biofertilizers. Biofertilizers Manual. Forum for Nuclear Cooperation in Asia. Japan pp. 112-115

[CR53] Świechowski W, Doruchowski G, and Trzciński P (2012) Effect of spray application parameters on viability of bacterium *Pseudomonas fluorescens* used as bio-pesticide in organic fruit production. In: Granatstein D, and Andrews P (eds.) Proc. II Int. Congress on Organic Fruit Research Symposium. ISHS, Leavenworth WA, USA, pp. 9

[CR54] Toro M, Azcón R, Barea JM (1998). The use of isotopic dilution techniques to evaluate the interactive effects of *Rhizobium* genotype, mycorrhizal fungi, phosphate-solubilizing rhizobacteria and rock phosphate on nitrogen and phosphorus acquisition by *Medicago sativa*. New Phytol.

[CR55] Vassilev N, Vassileva M, Fenice M, Federici F (2001). Immobilized cell technology applied in solubilization of insoluble inorganic (rock) phosphates and P plant acquisition. Bioresour Technol.

[CR56] Vassilev N, Medina A, Azcon R, Vassilev M (2006). Microbial solubilization of rock phosphate as media containing agro industrial wastes and effect of the resulting products on plant growth and phosphorus uptake. Plant Soil.

[CR57] Vassilev N, Vassileva M, Nikolaeva I (2006). Simultaneous P-solubilizing and biocontrol activity of microorganisms: potentials and future trends. Appl Microbiol Biotechnol.

[CR58] Vessey JK (2003). Plant growth promoting rhizobacteria as biofertilizers. Plant Soil.

[CR59] Wang X, Pan Q, Chen F, Yan X, Liao H (2011). Effects of co-inoculation with arbuscular mycorrhizal fungi and rhizobia on soybean growth as related to root architecture and availability of N and P. Mycorrhiza.

[CR60] Watt M, Kirkegaard JA, Passioura JB (2006). Rhizosphere biology and crop productivity—a review. Aust J Soil Res.

[CR61] Wu SC, Cao ZH, Li ZG, Cheung KC, Wong MH (2005). Effects of biofertilizer containing N-fixer, P and K solubilizers and AM fungi on maize growth: a greenhouse trial. Geoderma.

[CR62] Xie X, Yoneyama K, Yoneyama K (2010). The strigolacone story. Annu Rev Phytopathol.

